# Synthesis and Application of Cellulose-Polyethyleneimine Composites and Nanocomposites: A Concise Review

**DOI:** 10.3390/ma14030473

**Published:** 2021-01-20

**Authors:** Laura Riva, Andrea Fiorati, Carlo Punta

**Affiliations:** Department of Chemistry, Materials, and Chemical Engineering “G. Natta” and INSTM Local Unit, Politecnico di Milano, 20131 Milano, Italy; laura2.riva@polimi.it (L.R.); andrea.fiorati@polimi.it (A.F.)

**Keywords:** cellulose, nanocellulose, polyethyleneimine, biopolymer composites

## Abstract

Cellulose/polyethyleneimine composites have increasingly attracted the attention of scientific community, devoted to the design and development of new synthetic strategies and materials for different application fields. In this review, after introducing the main characteristics of the two polymeric components, we provide in the second section a critical overview on the main protocols for the synthesis of these composites, considering both the several cellulose sources and forms, and the different cross-linkers and cross-linking procedures developed for this purpose, outlining advantages and limits for the reported approaches. The last section analyses the principal results obtained in different application fields. A wide discussion is dedicated to the principal use of cellulose/polyethyleneimine composites as sorbents for water remediation from heavy metal ions and organic contaminants. Subsequently, we introduce the literature describing the use of these composites, functionalized appropriately, where necessary, as drug delivery systems, sensors, and heterogeneous catalysts for organic reactions. Finally, after a brief description of other random applications, we furnish a personal analysis of actual limits and potentialities for these systems.

## 1. Introduction

Polymeric composites are widely used as primary supporting structures in different fields due to their combination of excellent mechanical properties with low density [1,2]. In recent years, growing environmental awareness towards renewable resources and environmental issues has enhanced the interest in the development of engineered products made from natural resources, using natural fibers as an alternative to traditional synthetic ones in composite materials [3,4,5,6]. Among the natural polymers, cellulose represents the most abundant, renewable, and biodegradable source in the biosphere, making it a good alternative to petroleum-based materials [7].

Combination of cellulose or nanocellulose matrices with polyethyleneimines (PEI) has led to the production of a wide range of composites, which have found ample application in different fields. The potentialities of these hybrid systems have attracted more and more attention from the scientific community, and the number of publications in this field has progressively increased in recent years, with a significant leap forward in 2020, which accounts for 71 papers related to the synergic use of cellulose and PEI (source Scopus^®^ by Elsevier), mainly (but not exclusively) dedicated to the development of sorbent systems for water remediation. Being active in this research topic, we found it useful to critically analyze what has already been done, in order to lay out the bases for future developments.

In this review, after briefly introducing the two main components of these composites, we offer a critical overview of the main synthetic strategies to produce cellulose–PEI composites (Section 2), and an admittedly personal selection of the main application fields for these interesting materials (Section 3). Finally, we share with readers some criteria which, in our opinion, should be taken into consideration for future research in this promising field.

### 1.1. Cellulose

#### 1.1.1. General Overview

Cellulose is a linear macromolecule formed by *β*-1,4-linked D-glucopyranose rings. It is one of the most important structural elements in plants and bacteria, with the role of maintaining their inner structure [8]. Cellulose is colorless, odorless, tasteless, nontoxic, and exhibits intriguing properties such as excellent mechanical strength, biocompatibility, hydrophilicity, relative thermal-stabilization, high sorption capacity, and alterable optical appearance [9]. Moreover, it is insoluble in water and in most organic solvents and exhibits a chiral structure. The single polymer chains assemble into fibers through intermolecular hydrogen bonds and hydrophobic interactions. The terminal groups of each cellulose chain give directional asymmetry. The reducing end of the chain has a hemiacetal group, while the non-reducing end bears a pendant hydroxyl group [10]. Most of the polymer’s properties are related to its chain length (degree of polymerization). Cellulose from wood pulp has a chain length with a range between 300 and 1700 units, while cotton (and other plant fibers) and bacterial cellulose have a length in the range of 800–10,000 units [11].

**Figure 1:**
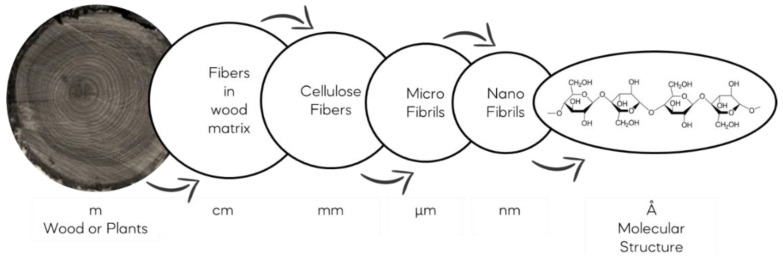
Inner structure of cellulose obtained from lignocellulosic biomass.

Cellulose is a straight chain polymer. This property allows the adoption of a quite stiff rod-like structure, facilitated by the equatorial conformation of the glucose residues. The bond between the units is made through condensation of the hydroxyl groups present on the monomer, forming chains with high tensile strength. Moreover, cellulose has a hierarchical structure where single chains are meshed into fibers (Figure 1) [12]. Cellulose derivatives are obtained through the selective conversion (full or partial) of the hydroxyl groups (-OH) by means of several reagents, obtaining materials characterized by different functionalities.

The most important source of cellulose is lignocellulosic biomass, which is composed of cellulose (30−50 wt.% [13]), hemicellulose (19−45 wt.% [14]) and lignin (15−35 wt.% [15]). All these components together form a hetero matrix that varies in its composition and structure as a function of the biomass source.

The architecture of the hierarchical structure of cellulose may be described as follows: the smaller components are linear glucan chains, which combine in crystalline cellulose nanofibrils 3–4 nm wide (30–40 cellulose chains each). Bundles of nanofibrils lead to the formation of microfibrils and, in turn, cell walls, fibers, and plant tissues [16]. The main cellulose sources include:

- agricultural residues;
- tree trunks and dead forest matter;
- cotton;
- energy crops;
- food waste;
- municipal and industrial biowaste such as used paper, carton, and wood from demolition sites.

Obviously, cotton and wood represent an important natural source of cellulose. However, the high and wide availability of wasted biomass should increasingly push things towards a circular economy route, making the production of cellulose composites sustainable, in order to minimize the impact on the environment by overconsuming natural resources. This criterion should also be taken into account for the production of nanocellulose.

#### 1.1.2. Nanocellulose

The cleaving of the hierarchical structure of cellulose fibers allows producing nano-sized cellulose (nanocellulose (NC)), which is characterized by at least one dimension under 100 nm. The unique characteristics of NC include thermal resistance, tensile and mechanical strength, a Young’s modulus in the range of 20−50 GPa, and a high and highly reactive surface area [17]. All these characteristics confer nanocellulose with promising properties, making it useful for several applications.

NC can be used to produce various high-value products with low environmental impact, and it can be produced industrially at a tons-per-day scale, following different and low-impact procedures [18].

NC can be obtained in different forms: cellulose nanocrystals (CNCs), cellulose nanofibers (CNFs), and bacterial nanocellulose (BNC). CNCs are nanometers long and show crystalline rod-like fragments as a result of acid hydrolysis. CNFs are produced by means of mechanical, chemical or enzymatic shearing techniques, deconstructing cellulose fibers into sub-structures with a nanometric width and a length in the order of micrometers [7]. While CNCs and CNFs are prepared via a top-down process, BNC can be produced through a bottom-up approach, using cultures of bacteria. However, if this latter approach from one side makes it possible to produce pure and crystalline nanocellulose, it still suffers not only from important limitations with respect to large-scale production, but also does not meet the criteria of valorization of biowaste, from which cellulose should be extracted and recycled.

Thanks to its surface functional groups, NC can be both grafted and cross-linked, opening to the formation of highly porous and mechanically strong composites. Moreover, it can be considered a suitable and sustainable reinforcement for bio/nanocomposites [19,20]. The addition of rigid particles to polymer matrices imparts several positive effects, e.g., improved stiffness, reduction in the thermal expansion coefficient, and increase in creep resistance and fracture toughness. NC-reinforced polymers have been found to be more efficient, with better mechanical properties than those crafted using conventional micro- or macro-composite materials.

**Scheme 1:**
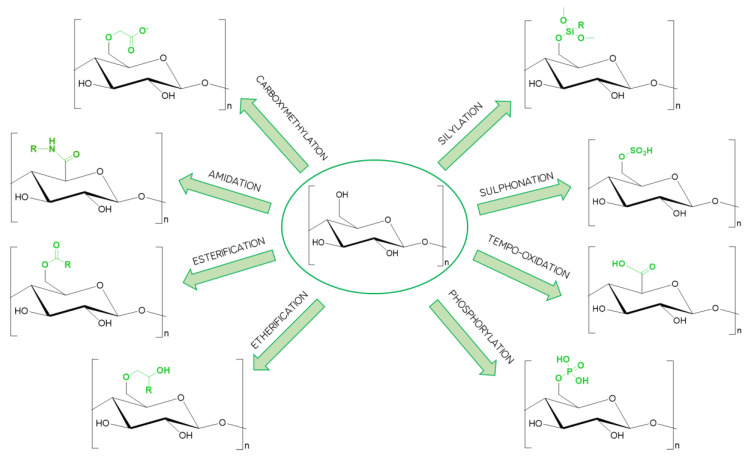
Different chemical functionalizations of cellulose at the C6 position.

A limit to some nanocellulose applications is represented by its intrinsic hydrophilic nature. This restraint can be overcome using surface modification techniques. Carboxymethylation, amidation, esterification, etherification, silylation, sulphonation, and phosphorylation are just some examples of the possible surface modifications aiming to the alteration of the properties and to the extension of potential NC applications (Scheme 1) [21].

### 1.2. Polyethyleneimine

**Scheme 2:**
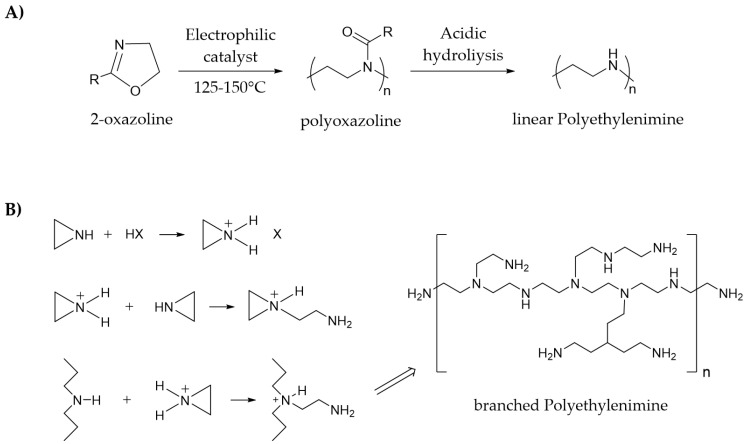
Synthesis of (**A**) linear PEI [25] and (**B**) branched PEI [26].

Polyethyleneimine (PEI, Scheme 2A), including branched polyethyleneimine (bPEI, Scheme 2B) is a polymer bearing a huge number of amine groups [22], with an amine density significantly higher when compared with most commercial cellulose-fiber coupling agents [23,24].

This characteristic confers several advantages to this family of polymers. On one hand, the amino groups are ideal functional units for promoting the covalent grafting on cellulose fibers and their cross-linking in reinforced composites. On the other hand, they can significantly extend the interaction capacity of the cellulose–PEI composite with different matrices or molecules, expanding the possible fields of application of resulting materials, as will be discussed in Section 3. Moreover, the presence of amino units also opens the way for further functionalization of (b)PEI polymers, before or after their formulation in the cellulose-based composites, with the consequent opportunity to introduce additional active sites for specific uses [27,28].

On the other hand, potential cytotoxicity and ecotoxicity of this polymer should be considered in order to guarantee the safety of the new devices, especially when final application would see direct contact with living beings and the environment. (b)PEI is considered a gold standard as cationic non-viral vector for gene-delivery therapy, as it exhibits good transfection performance [29,30]. However, as expected for macromolecules bearing a high number of positively charged moieties, a moderate but not neglectable cytotoxicity is also associated with this class of cationic polymers, directly proportional to the molecular branching and charge density, and possibly reduced by proper functionalization [31].

Moreover, by considering that one of the main applications of cellulose–PEI systems is in the field of water remediation, ecotoxicity of (b)PEI should be also considered. As an example, Malysheva and co-workers reported in 2016 that the coating of silver nanoparticles with bPEI significantly increase their association with algal cells [32]. This observation should drive the design and optimization of cellulose–PEI composites, in order to provide stable systems and to avoid the release of this cationic polymer in the environment. This issue will be addressed in more detail in Section 3.1.1.

## 2. Cellulose–PEI Composites: Synthetic Strategies

### 2.1. Cellulose Sources

As mentioned previously, cellulose for composite synthesis can be derived from a variety of sources, as summarized in Table 1.

Cellulose powder is the most used source, due to its easy availability on the market, its low cost, and its easy handling. Cellulose powder has hydroxyl groups on the surface and is therefore easily dispersible in polar solvents such as water [33,34,35,36]. Another form of cellulose specifically used for the synthesis of composites is microcrystalline cellulose [37,38], which has similar characteristics to cellulose powder, but has a larger exposed surface area, thus increasing its reactivity in the chemical environment and facilitating interaction with the other components of the mixture. For the same reason, nanocellulose has also been used for the synthesis of cellulose-based composites [39,40]. However, the versatility of cellulose also allows the use of immediate and inexpensive cellulose sources, as reported by Setyono et al., and by Zhao J. et al., who used filter paper and sheets of paper, respectively, as cellulose sources for the synthesis of bPEI-composites [41,42] or by He and co-workers, who proposed the direct grafting of simple medical degrease cotton [43].

### 2.2. Cross-Linking Strategies

**Scheme 3:**
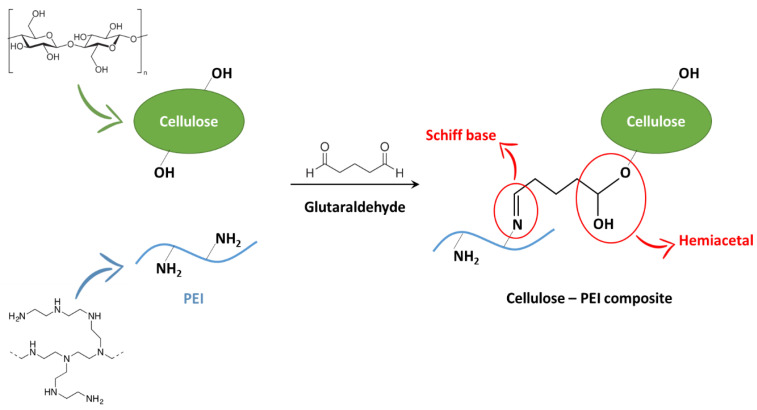
Cross-linking between PEI and cellulose in presence of GAL.

The synthesis of cellulose–PEI composites can be achieved by following different routes. Starting from cellulose sources without pre-functionalization of the fibers often necessitates the use of proper cross-linkers in order to obtain stable and resistant composites. On the contrary, when operating in the presence of pre-functionalized cellulose the direct cross-linking between the two building blocks is sometimes possible under proper conditions, without requiring the use of additional cross-linkers. In the first case, the most frequently used cross-linkers are glutaraldehyde (GAL) and epichlorohydrin (EPI). The use of GAL involves cross-linking between (b)PEI and cellulose through the formation of a Schiff base and a hemiacetal, as shown in Scheme 3. The reaction can be conducted one pot through simple stirring in polar solvents [35,38], and it is possible to operate even at room temperature. However, by increasing the reaction temperature, it is possible to increase the value of the cellulose/PEI ratio, obtaining a more stable product thanks to the higher amount of the cellulosic polymer [33].

**Scheme 4:**
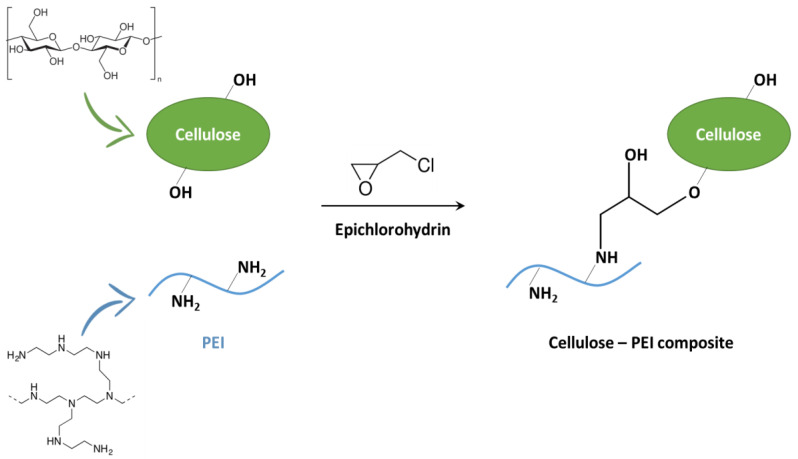
Cross-linking between PEI and cellulose in the presence of EPI.

The use of EPI instead requires heating to form stable composites. The mechanism of reaction is described in Scheme 4. As reported by Ge et al. [28], a treatment at 60 °C in oven after mixing the components at room temperature is necessary in order to promote the cross-linking, while He et al. performed an analogous approach by stirring the reagents’ mixture in a round-bottom flask at 90 °C in a 2 M NaOH aqueous solution [43]. An alternative synthetic approach consists into promoting epichlorohydrin-mediated cross-linking by microwave irradiation [40]. This protocol allows a significant reduction in the reaction time and guarantees a more homogeneous heating of the mixture.

While these synthetic strategies could be considered simple and, even in the case of EPI, operable under relatively mild conditions, we should not neglect the fact that both GAL and EPI have been reported to be highly toxic for humans and animals [44,45]. By designing a new material, and a cellulose–PEI composite as well, it should be possible to address the challenge of health and environmental safety from the very beginning, focusing not only on the optimization of performances for the application of interest, but also on overcoming all the potential side effects that the system might imply both during the production process and in the environment of its final use.

**Scheme 5:**
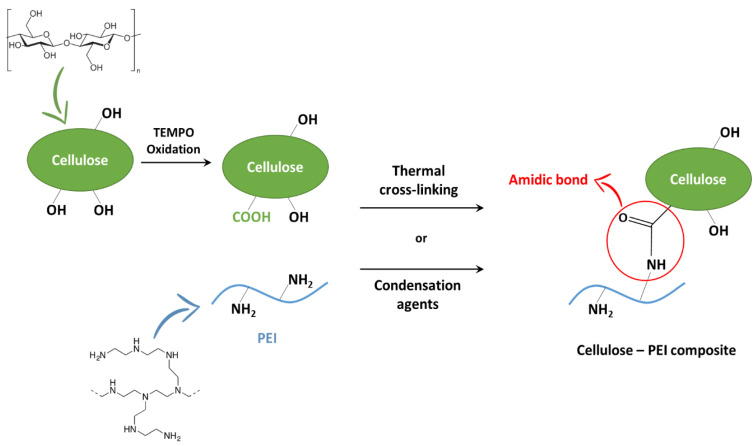
Synthesis of the cellulose–bPEI composites without the aid of a cross-linker.

Consequently, if cellulose pre-functionalization an occur under sustainable and scalable conditions, and the cross-linking with PEI can take place without the aid of a cross-linker (Scheme 5), this approach should be selected as the preferable one, as this would avoid the use of potentially toxic additives.

A significant example is represented by the cellulose–bPEI nanosponges proposed by our group and obtained by mixing (2,2,6,6-Tetramethylpiperidin-1-yl)oxyl (TEMPO)-Oxidized Cellulose NanoFibers (TOCNF) and branched polyethyleneimine. TOCNF are extremely cheap and easy to synthesize by a simple oxidation process based on the TEMPO/NaBr/NaClO system [46]. The oxidation results in the partial conversion of the alcoholic moieties at the C6 position of the glucopyranose units to the corresponding carboxylic acids. Resultingly, these nanofibers are extremely flexible, and possess a good surface area and an excellent reactivity due to the introduction of carboxylic units. Our group suggested the possibility of cross-linking TOCNF and bPEI 25 kDa by means of a freeze-drying process, followed by heating in oven up to 100 °C. The resulting composites, despite the absence of additional cross-linkers, resulted stable even in water, thanks to the formation of amide bonds between the two components during the thermal treatment [47]. Interestingly, their mechanical properties can be further enhanced by the introduction of a natural co-cross-linker, citric acid, with the aim of further stabilizing the structure of the composite by promoting the formation of additional amide bonds with the free amines of bPEI [48]. This approach made it possible to obtain cellulose nanosponges (CNS), so called for their inner nanostructure and nanoporosity, as revealed in recent studies [49,50]. Reticulation in the absence of additional cross-linkers could also occur when following processes different from that of thermal treatment, such as through the use of condensation agents such as EDC ((1-ethyl-3-(3-dimethylaminopropyl)carbodiimide). The latter is able to promote amide bond formation between the amines of PEI and the carboxyl groups of TEMPO-oxidized cellulose. The advantage of this synthetic route relies on the possibility of operating at room temperature, without resorting to higher temperatures, as reported in the work of Swasy and co-workers [51]. Which of the two strategies should be considered the preferred one is hard to say. Once again, the thermal approach allows to reach the final goal (condensation), without requiring further additives as in the case of the use of condensation agents. However, it is a matter of fact that this process, at least in its actual design, which implies a freeze-drying step, is energy intensive, and this aspect has an indirect impact on the life cycle assessment of the overall synthesis [52].

Another reported route for cellulose functionalization takes advantage of the reactivity of silane derivatives bearing active sites, which are able to promote the formation of covalent bonds between cellulose hydroxyls and the amino groups of PEI. This grafting strategy permits the introduction of moieties, such as epoxy groups, that can in turn react with the amines present on PEI.

**Scheme 6:**
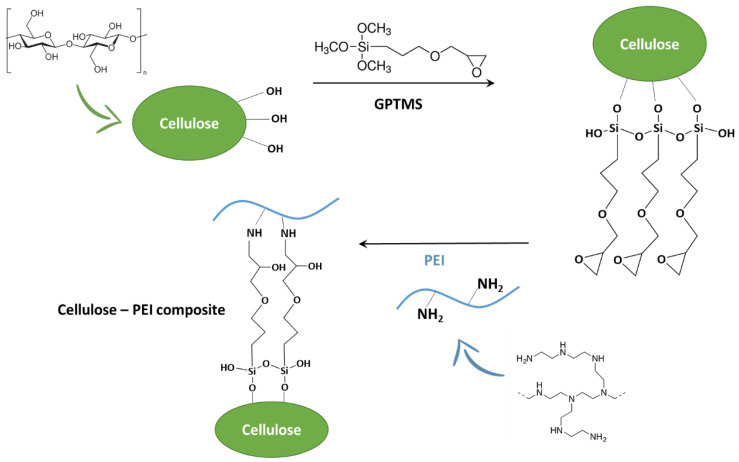
Cross-linking between PEI and cellulose in presence of GPTMS.

This approach allows operation under mild conditions, as all reactions usually occur at room temperature using water as solvent, the latter generally removed by freeze-drying at the end of the synthesis. This is the case of γ-(2,3-epoxypropoxy)propyltrimethoxysilane (GPTMS), which has been widely used for the preparation of cellulose–PEI xerogel scaffolds (Scheme 6) [53,54,55]. It is interesting to note that, except for the chemical and mechanical stability, GPTMS, as well as the other cross-linkers, seems to confer no additional functional properties to the composites.

Another interesting approach is the one recently reported by Kriechbaum and co-workers. They described the production of an aerogel by adsorbing PEI on TOCNF and then promoting the covalent cross-linking by means of a Michael-type reaction between oxidized catechol-containing tannin and PEI amines, followed by a drying step via evaporation [56].

## 3. Cellulose–PEI Composites: Fields of Application

The combination of these two building blocks, cellulose and PEI, leads to composites with specific characteristics. Cellulose makes them mechanically stable, also when exposed in water for long time. PEI, thanks to the numerous amine groups present on the surface, gives the composites particular reactivity and chelating properties, together with a marked alkalinity. All these characteristics have made these materials extremely attractive for being used in different fields, including environmental decontamination from heavy metals and organic contaminants, recovery of Rare Earth Elements (R.E.E.), drug release, sensing, heterogeneous catalysis, and other applications that will be discussed in detail in the next paragraphs.

### 3.1. Water Remediation

#### 3.1.1. Heavy Metal Ions Removal

Water pollution by transition metals is a grave menace to public health, as levels of heavy metals, such as As(III-V), Cu(II), Zn(II), Cd(II), Ni(II), Cr(III), Cr(VI) and Pb(II), that are too high can cause gastrointestinal, liver, kidney damage and intravascular hemolysis. Many attempts have been made to find a solution to this problem and in many cases natural polymer-based composites have offered good strategies to face this issue. Considering all the properties discussed above, cellulose–PEI composites result in good candidates for the sorption of contaminants for water remediation. Thanks to the massive presence of primary amines on (b)PEI, metals can be easily adsorbed by these composites through chelation phenomena. Indeed, this represents the most relevant application for which cellulose–PEI composites are designed and used.

Different aspects can modulate the removal efficiency, strictly related to the final material conformation and composition, which determine the availability and exposition of active chelating sites. From this point of view, the nano-structuring of the material, starting from CNC and CNF as building blocks, would guarantee a higher surface area and a more efficient detection of transition metal contaminants. A general overview of cellulose–PEI composites for the removal of heavy metals is provided in Table 2.

Among all the metal ions of interest, Cu(II) has been widely taken to be a reference in adsorption studies since it is well adsorbed and the sorption is easy to detect, also by simple UV analysis. However, precisely because it is quite easy to be adsorbed due to its high capacity to undergo chelation, a decontamination performance analysis just based on this ion should be considered limiting.

Ample focus on this metal has been given by Ge and co-workers, who demonstrated the efficiency of their cellulose–bPEI composites, produced with EPI as cross-linker, in adsorbing Cu(II) from aqueous solutions [34]. For the above-mentioned reasons, cellulose–PEI composites have often proven to be selective towards Cu(II). A striking example is the work by Melone et al., where the sorption efficiency of CNS previously described against metals such as Cu(II), Co(II), Ni(II) and Cd(II), was tested. The materials were well suited for the adsorption of all metals considered, but through competition tests in multi-contaminated solutions, CNS showed a marked selectivity towards Cu(II) ions [47]. Another metal efficiently adsorbed through PEI- cellulose-based composites is Arsenic. Deng et al. reported that this metal in its two forms, As(III) and As(V), was well adsorbed by cellulose fiber-based and hyperbranched polyethyleneimine composites [57].

One of the factors to consider for the adsorption of metals, especially Cu(II), Pb(II) and Cr(II), is the pH. As reported in several papers, the adsorption of these metals occurs optimally at pH between 4.5 and 7.0 [41,58]. For the adsorption of Arsenic, too, especially As(V), the pH is a very impacting factor, with a maximum uptake amount at pH 4.0 [57]. This is in part surprising, as the chelating action should be favored at alkaline pH, which guarantees deprotonation of amine groups.

A feature that can often be found in this category of composites, and represents a considerable advantage, is the possibility to regenerate them by simple washing with diluted acid solutions and reuse the system several times. The mainly used acids are HCl, as in the case of Tang et al. [59], and ethylenediaminetetraacetic acid (EDTA), as in the work of Wang et al. and Jin et al. [58,60]. With both solutions, an efficient regeneration of the material can be obtained, with a minimum reduction of the adsorption capacity of the composite.

An important drawback for most of the cellulose–PEI composites for water decontamination reported in the literature consists in the lack of evaluation in realistic scenarios, meaning at least in more complex artificial matrices. In fact, in most cases, the remediation action of the materials has been tested for mono-contaminated milliQ^®^ (Merck KGaA, Darmstadt, Germany) solutions, that is, under conditions far from their potential application in the field. Neglecting this aspect has a double consequence. On the one hand, we entertain the risk of underestimating the interfering role of other organic and inorganic components in the interaction between composite and contaminant. On the other hand, we miss estimating the eco-safety of the material solution once contacted with a real aqueous ecosystem [61].

**Table 2:** General overview of cellulose–PEI composites for adsorption of heavy metals from water. The table shows the values of reported adsorption capacity for each composite expressed in mg·g^−1^.

Composites	Cross-Linker	Cu(II)	As(III)	As(V)	Zn(II)	Cd(II)	Ni(II)	Pb(II)	Co(II)	Cr(III)	Cr(VI)	Fe(III)	Reference
**PEI-functionalized paper**	**GAL**	435	-	-	-	370	208	-	-	-	-	-	[41]
**PEI-modified microcrystalline cellulose**		-	-	-	-	217	-	357	-	-	-	-	[38]
**PEI-modified TOCNF**		53	-	-	-	-	-	-	-	-	-	-	[62]
**PEI-cellulose aerogel beads**		-	-	-	-	-	-	-	-	-	229	-	[63]
**Coffee-derived cellulose–PEI membranes**		-	13	46	-	-	-	-	-	-	-	-	[64]
**PEI-nanowood**		93	-	-	-	-	-	-	-	-	-	-	[65]
**bPEI-modified CF**	**EPI**	-	56	94	-	-	-	-	-	-	-	-	[57]
**PEI-cellulose hydrogel**		286	-	-	-	-	-	-	-	-	-	-	[34]
**Porous spheres**		-	-	-	-	-	-	-	-	84	-	377	[43]
**CMC-PEI composites**	**GMA ^a^**	102	-	-	-	-	-	-	-	-	-	-	[37]
**CNF-based aerogels**	**None**	103	-	-	-	-	-	-	-	-	-	-	[59]
**CNF-PEI aerogels**		175	-	-	-	-	-	357	-	-	-	-	[66]
**CCN-PEI composites**		-	-	-	-	-	-	-	-	-	358	-	[39]
**PEI-BC composites**		148	-	-	-	-	-	141	-	-	-	-	[58]
**PEI-BCNF membranes**		90	-	-	-	-	-	130	-	-	-	-	[60]
**TOCNF-bPEI aerogels**		84	-	-	32	13	10	-	9	40	-	-	[47,67]
		194 ^c^	-	-	125 ^c^	84 ^c^	80 ^c^	-	-	94 ^c^	-	-	[68,69]
	**TMPTAP ^b^**	485	-	-	-	-	-	-	-	-	-	-	[70]
**CNF-supported cryogel**	**GPTMS**	129	-	-	-	-	-	-	-	-	-	-	[71]

^a^ GMA: Glycidyl methacrylate. ^b^ TMPTAP: Trimethylolpropane-tris-(2-methyl-1-aziridine) propionate. ^c^ Expressed values indicate adsorptions made in artificial seawater.

**Scheme 7:**
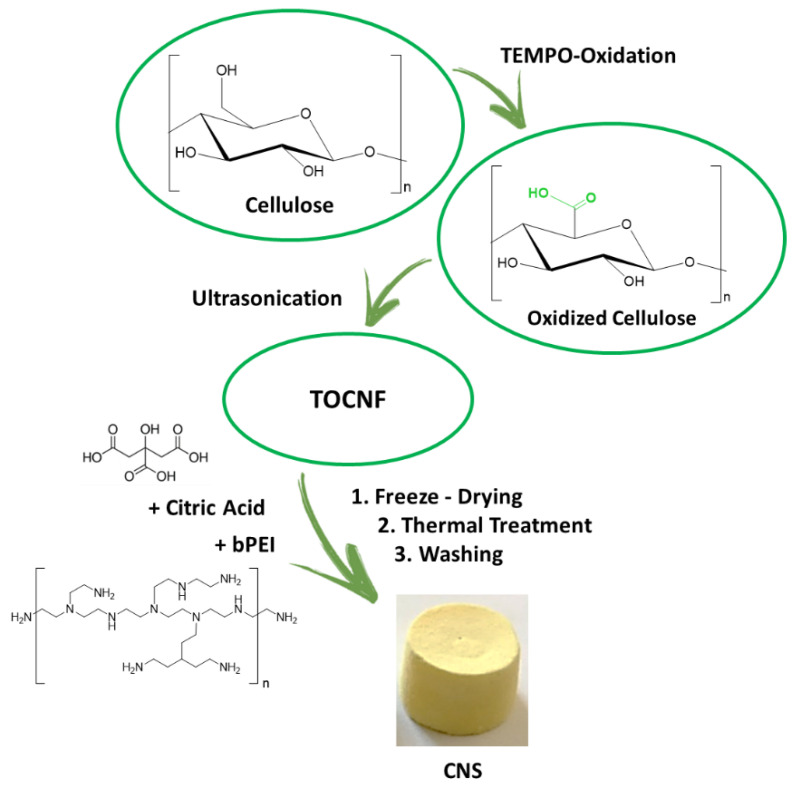
Optimized synthesis of CNS, with the addition of citric acid as a co-cross-linker and an increased purification process.

The CNS previously described could represent an interesting case study. Very recently, as a result of testing their adsorption efficiency in artificial sea water [47], we were pushed to re-consider the synthetic protocol and the final formulation, by following an eco-design approach, that is, by evaluating step by step the ecotoxicological impact of our composite (Scheme 7).

In fact, while the adsorption efficiency was confirmed to be quite high even in this complex matrix, by testing our original formulation, we observed toxicity towards the model marine microalga *D. tertiolecta*, as shown by a consistent growth rate reduction [68]. We verified that this result had to be ascribed to a partial release of toxic bPEI in the water medium, in line with what was previously reported and discussed in Section 1.2 [31,32]. We overcame this issue by better fixing this polymer in the cellulose matrix by addition of citric acid as a cross-linking agent. Moreover, we modified the synthetic protocol to minimize the excess of bPEI and optimize the purification strategy, confirming the efficacy of our action by conducting new in vivo exposure tests also with mussels.

Moreover, the new composite has been further tested for its remediation action in realistic media, both in fresh water mono-contaminated with Cd(II) [72] and in sea-water mono-contaminated with Zn(II) [69]. In both cases, once again, the remediation approach was validated by in vivo tests.

These findings reinforced our conviction that, to better estimate their impact as efficient sorbent systems, most of the products reported in the literature should be re-considered from an eco-safety point of view, and possibly discarded or re-designed once tested in real, or at least realistic, scenarios.

As a last aspect to be addressed in this section, the increasing demand for Rare Earth Elements (REEs), due to their use in various technological applications, has stimulated the development of new approaches for efficient REEs separation and recovery, with particular attention to sustainability. Additionally, in this case, cellulose–PEI composites have found successful application. Zhao et al. reported the synthesis of a CNC-PEI system for the recovery of REEs, by promoting the cross-linking of the two components in aqueous solution, without further addition of toxic cross-linkers [40]. The adsorption of all the REEs studied (La(III), Eu(III), and Er(III)) was very fast in the initial phase of the process, with a 45% of the maximum uptake registered in the first 5 min.

Hong and co-workers reported the production of a nanocellulose–PEI composite, cross-linked by means of GAL, and have exploited its function for the recovery of platinum from a catalyst leaching solution [73]. The authors pointed out the importance of the cellulose sources, as they have observed that the cross-linked PEI density, and consequently the adsorption performances, depends on the nanocellulose zeta potential and surface area. By this deep investigation, they were able to identify a composite possessing a Pt adsorption capacity up to about 600 mg·g^−1^. More recently, the same authors have also proved the selectivity and high capacity of Pt (120.2 mg·g^−1^, 86%) and Pd (26.5 mg·g^−1^, 74.2%) adsorption by these composites, by operating on a simulated automobile waste containing also other metals (Ni, Fe, Mn, and Cr) [74].

#### 3.1.2. Organic Contaminants Removal

Nowadays, over 100,000 dyes are commercially available. Synthetic dyes are widely used in several industrial production chains, including cosmetic, pharmaceutical, food, rubber, paper and textiles. During the washing process, a certain fraction of dyestuffs unavoidably ends up in the wastewater. This waste has to be removed before the release of the water in the ecosystem, due to the potential toxicity and negative effects on the aquatic environment.

Examples of the removal of these contaminants have been reported using different cellulose–bPEI composites, in the form of membranes [75] or aerogels, and obtained either in the presence [33] or in the absence [36] of cross-linkers. Aerogels have been synthesized following different protocols. In particular, Guo and co-workers used GAL as cross-linker, while also CNS produced according to our thermal method in the presence of citric acid were shown to be effective for organic dye adsorption [35,76]. Although these two approaches provide similar systems and, in both cases, good sorption performances, a comparison in terms of sustainability of the synthetic procedures should be discussed. GAL is a toxic additional ingredient in the formulation, while cross-linking by thermal treatment could be considered to be more easily scalable and less environmentally impactful. However, a more detailed investigation should be conducted, as a Life Cycle Assessment (LCA) at laboratory scale recently revealed how the CNS synthetic procedure still partially suffers from a relatively high energy consumption impact, due to the freeze-drying step [52].

Table 3 summarizes the most investigated dyes for testing the capture capacity of cellulose–PEI composites. The most common ones are in an ionic form. Removal efficacy has been demonstrated for both cationic and anionic ones, even if the best performances were observed for the latter dyestuffs. This evidence was ascribed to an electrostatic interaction between the cationic charges of the amino groups of bPEI and the negative charges of anionic dyes, with a sorption efficiency depending on the charge present both on the surface and on the contaminant dye.

In addition to electrostatic interactions, when composites present a high nanoporosity, it is necessary to consider other factors, including the compatibility between the size or volumetric hindrance of the molecule to be adsorbed and the dimension of the sorbent pores. We observed a similar behavior when using our CNS. Molecules with a lower molecular weight such as Orange Sodium Salt II are adsorbed more efficiently than larger ones, such as Cibacron Brilliant Yellow, even if the latter have a higher number of sulfate groups, and should therefore establish a stronger electrostatic interaction with the sorbent.

Kinetic and isothermal studies [76] have shown that our CNS are comparable from a performance point of view with activated carbons [77], which are the most used porous materials for this type of application. Moreover, these composites could be regenerated and reused several times after treatment with diluted alkaline solutions [76], while activated carbons regeneration requires high temperature thermal treatments [78].

CNS was shown to be also particularly efficient in the adsorption of other emerging organic contaminants, including model phenols, industrial precursors of fungicides and pesticides, and amoxicillin, a representative example of antibiotic contaminants [47].

Finally, very recently, Swasy and co-workers reported the effective action of CNC-PEI composites, prepared using EDC as cross-linker, in promoting the degradation of three different pesticides (malathion, deltamethrin, and permethrin). In this case, while degradation into lower molecular weight by-products resulted particularly high for all the three target contaminants, more attention should be paid on the fate and the potential toxicity of the resulting molecules, as the action mechanism does not provide a complete demolition of the organic targets [51].

### 3.2. Drug Release

Despite significant improvements in smart drug delivery, the search for drug carriers with low cost and long-term sustainable release still presents an enormous challenge. Among cellulose–PEI composites, aerogels, which have received wide attention due to their high porosity, specific area and low density [79], have proved to be the most suitable in this kind of application. The combination of a porous structure with a good mechanical stability, and the presence of plenty amino-groups makes cellulose–PEI aerogels potentially useful as drug reservoirs for the release of active principles [80]. To prove the potential of these aerogels as drug carriers, the factors affecting aerogel drug loading were discussed. The selected drugs are molecules extremely widespread in the treatment of common diseases, such as sodium salicylate and ibuprofen as active principles for the treatment of inflammatory disorders and amoxicillin as antibiotic molecule.

Fiorati and co-workers focused on ibuprofen and amoxicillin as target molecules, and CNS described in Scheme 7 as aerogel models. Adsorption experiments were performed by introducing a single CNS in 20 mL of amoxicillin or ibuprofen methanol solution at different concentrations and at 25 °C. The loading on CNS was determined by measuring the lowering in drug concentration in solution after 4 h with a UV-spectrophotometer. These experiments evidenced high adsorption capability, essentially driven by the interaction between the carboxylic acid sites of the target molecules and the amines present on the bPEI-TOCNF composite. The release kinetics for amoxicillin and ibuprofen were investigated in phosphate-buffered saline at pH 7.0, while the release of the antibiotic was also carried out at pH 2.0 in order to simulate the highly acidic medium typically found in the stomach environment. The release performance was good for both active principles and in both conditions [48].

Yan and co-workers studied the adsorption process of sodium salicylate in the presence of similar systems, by investigating different parameters, in particular initial concentration of the drug and pH influence on the process. The best adsorption conditions were established to be at a concentration of 1.0 mg·mL^−1^ of sodium salicylate at a pH value of 3.0. The release performances were studied in function of pH and temperature. The best conditions found for the release process were neutral pH and 37 °C temperature, perfectly fitting with the physiologic conditions of the human body [81].

### 3.3. Selective Sensing

Systems for the recognition and sensing of analytes in solution are of particular interest for the industrial and research world. As an example, since anionic species are commonly involved in many biological processes related to human health, they can be considered as key-indicators for industrial pollution or geogenic hazardous contamination [82,83,84]. The development of eco-compatible selective sensors can be considered highly relevant for different fields of applications, especially if one of the building blocks is an eco-compatible matrix like cellulose and the detection system is based on a fast colorimetric sensing by the naked-eye, without requiring complex techniques which could negatively affect the costs of detection.

In this context, fluoride anions have become a subject of interest because they are potentially toxic to human health at low concentration [85]. A cellulose–bPEI sensor aerogel was reported by Melone et al. [86]. TOCNFs were thermally cross-linked with bPEI functionalized with *p*-NO_2_-phenyl urea as a sensing unit to obtain a sensor system selective towards fluoride anions in DMSO solutions up to a concentration of 0.5 M. Our research group improved these performances by replacing the sensor unit linked to bPEI with three other sensor molecules (mono and bis-naphthalene di-imides and bis-perylene di-imide). These units are able to undergo electron transfer with fluoride anions, making it possible to reach a detection limit one order of magnitude lower when operating with the bis-perylene di-imide. Other remarkable aspects of these systems are the selectivity towards fluoride anions, excluding other interfering anions such as monobasic phosphate and acetate, and the reusability after mild washing with polar solvents [87]. However, it should be noted that these systems are not able to work in the presence of water since the mechanism of interaction between the fluoride anion and the sensor molecules requires dry polar aprotic solvents [88,89].

Takahashi and co-workers have recently reported the design of cellulose-based voltammetry sensors for diol and polyol compounds, like 3,4-dihydroxyphenylalanine (*L*-DOPA) [90,91]. In a first work, the authors coated a gold electrode with films composed by carboxymethylcellulose, cellulose, and PEI, following a layer-by-layer approach. The electrode was then imbibed with Alizarin red S, which confers to the system a redox activity exploitable for sensing of polyol compounds. Its electrochemical properties could be tuned in the presence of phenylboronic acid (PBA) [90]. The same system was later improved by the same group by grafting the PBA directly on the PEI, in order to enhance the sensing performances of the system [91].

Another interesting example of the importance of nanocellulose–PEI composites in the field of sensing can be found in the contribution of Zhang and co-workers, reporting the synthesis of gold nanoparticles–BNC composites. In this system, PEI has the double role of reducing agent for the synthesis of the gold nanoparticles (GPs) and linking agent between GPs and BNC. The obtained nanocomposite was employed as support for the horseradish peroxidase obtaining a biosensor with a detection limit lower than 1 µM. The same support could be exploited for the immobilization of other enzymes for heterogeneous catalysis [92].

### 3.4. Heterogeneous Catalysis

A field of great interest for research and industry is certainly catalysis, especially heterogeneous catalysis, which has numerous advantages over homogeneous one. The most remarkable ones are surely the possibility of easy recover and reusability of the catalyst, with a consequent simpler isolation of the final products, and the duration of the catalyst, with a considerable reduction in costs. Micro- and nanoporous materials have been widely used as heterogeneous catalysts [93], due to their significant catalytic efficiency towards final products, which has to be ascribed to a large surface area. In this context, cellulose and PEI are often employed together for enzyme immobilization, obtaining heterogeneous catalysts which can be employed for a wide number of applications [84,86,87]. However, a deep discussion of this topic is beyond the scope of this review.

**Scheme 8:**
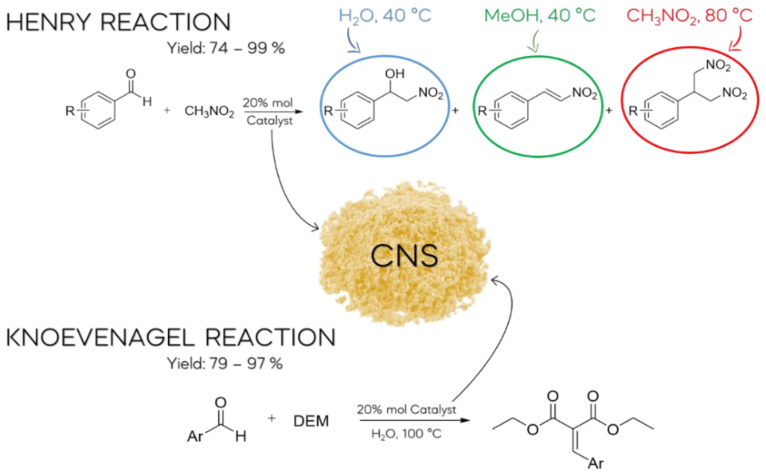
Henry and Knoevenagel reactions catalyzed by CNS [94].

The combination of nanocellulose and PEI may create three-dimensional composites with micro- and nanoporous patterns, suitable for catalytic applications. This is the case of CNS (Scheme 7), which, while developed for water remediation purposes, were shown to be particularly effective when tested as heterogeneous catalysts for the promotion of amino-catalyzed organic reactions, namely Knoevenagel and Henry reactions (Scheme 8) [94]. Interesting, the selectivity observed in these processes is quite different from that obtained by operating with bPEI in solution. This result opens the way towards the design of new catalytic systems, for example in combination with metal ions, which could be advantageous not only for overcoming the issue of the recovery and reuse of the polymeric catalyst, but also for providing new and alternative synthetic strategies for the obtainment of unconventional products.

**Scheme 9:**
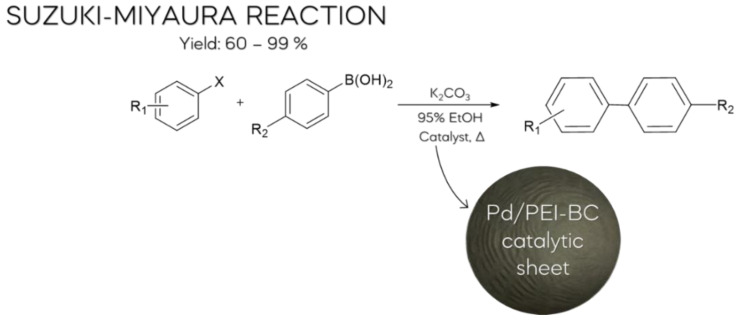
Suzuki–Miyaura reaction catalyzed by Pd/PEI-BC catalytic sheets.

Another recyclable dip-catalyst was reported in 2018 by Xiang and co-workers. In this work, BNC was oxidized with sodium periodate in order to introduce some aldehydic carbonyls, which were shown to be able to react with PEI forming the corresponding Shiff base. The latter was consequently reduced to a secondary amine by means of sodium cyanoborohydride. The obtained nanocomposite was then decorated by in situ synthesis of palladium nanoparticles, and then added to plant fibers, affording a dippable catalyst for Suzuki–Miyaura reaction. The catalyst guarantees a high reaction rate, a high turnover frequency, and can be reused up to 26 times without losing its catalytic efficiency (Scheme 9) [95].

Bacterial cellulose–PEI membranes, purposely designed for the removal of Cu^2+^ and Pb^2+^ heavy metal ions from aqueous solutions, were reused in an alternative way after the remediation action, in place of simple regeneration. In particular, Cu^2+^ loaded membranes were immersed in a hydrazine hydrate solution to reduce the copper ions and then put into methylene blue aqueous solution with the addition of NaBH_4_, obtaining an effective catalytic effect for the reduction of methylene blue [60]. A similar approach was reported by Zhang and co-workers, who decorated CNFs-PEI membranes with silver nanoparticles and then exploited their catalytic activities for continuous discoloration of aqueous cationic and anionic dye solutions [96].

In both cases, it is important to mention that the systems can be used several times as a catalyst, thanks to a simple washing in water, which makes them ready for the next reaction with no obvious worsening in performance.

### 3.5. Other Applications

Besides the main application fields previously discussed, cellulose–PEI composites have found some other technological uses addressed to different fields, ranging from the bio-building sector to biomedical applications and multifunctional wearable materials.

Sehaqui and co-workers reported the direct adsorption of CO_2_ from air onto a foam composed by TOCNF and PEI obtained by a freeze-drying process. The resulting xerogels possessed a porosity above 97% and a specific surface area in the range of 2.7−8.3 m^2^·g^−1^. The authors observed that CO_2_ capacity varies in function of the PEI content and the relative humidity. The kinetics of the process were fast (adsorption half time of about 10 min) and that the material could be reused up to five times [97].

In the work by Lee et al., transparent cellulose nanopapers were synthesized through a vacuum filtration-assisted layer-by-layer deposition method. These cellulose nanopapers, treated with a bPEI solution for the creation of a positively charged side and with a polyvinyl phosphonic acid solution for the formation of a negatively charged side, showed significantly enhanced flame-retardant properties confirmed by pyrolysis combustion data and flammability tests [98]. Similarly, Pan and co-workers reported a CNFs-based composite where the cellulosic layer was covered with two bilayer coatings composed by PEI, melamine, and phytic acid. The layer-by-layer self-assembly approach allowed to reduce the flammability of polyvinyl alcohol. In this composite CNFs act as carbon source, phytic acid as acid source, while PEI and melamine serve as blowing agents [99].

Antibacterial properties of bacterial cellulose and polyethyleneimine-based hybrid hydrogels were reported by Wahid and co-workers. These hydrogels were prepared using EPI as coupling agent and were established to be thermally stable and moldable, and to have mechanical properties strictly dependent on the PEI-content. Their antibacterial properties, which resulted in be also dependent on PEI content, were investigated against *S. aureus* and *E. coli* bacteria, using agar well diffusion and colony forming unit methods [100].

Finally, CNF-PEI aerogels can also undergo further modification in order to obtain multifunctional materials. This is the case of the composite material proposed by Wang et al., who reported a cellulose/polypyrrole/polyurethane composite that is flexible, waterproof, breathable and, thanks to its Joule heating performance, is proposed as a promising smart wearable device and personal thermal management system [101]. Cross-linking of CNF with PEI by means of GPTMS, as described in Section 2.2, afforded an aerogel on whose surface underwent an in situ polymerization of polypyrrole, followed by a coating with polyurethane and final addition of fluorinated agents.

There are several other examples of the combined use of cellulose and PEI in composites. However, in most of these cases, one of these two polymers, most often PEI, is not the main component of the final material, but is rather used as an adhesive or cross-linking agent. For this reason, these solutions are beyond the scope of the present review. Just as an example, we can mention here the deposition of graphene oxide/carboxymethyl cellulose composite films on AZ31 magnesium alloys, following a layer-by-layer assembly method in the presence of PEI, to provide an enhanced protective performance against corrosion [102].

## 4. Conclusions and Outlooks

The continuous increase in the number of research reports on the design, synthesis, and application of cellulose–PEI composites in the last decade clearly demonstrates the potentials and versatility of these systems. Many of these applications are related to the issue of water purification and, more generally, environmental remediation. The accessibility of a high number of both amino groups on PEI, and alcoholic, carbonyl and carboxylic groups on cellulose fibers and nanofibers, with the consequent possibility of introducing selected functional groups or nanoparticles, opens the way for the fabrication of more sophisticated and functionalized systems, capable not only of providing higher contaminant removal performances, but also of providing these biomass-based materials as candidates for sustainable solutions for a wider range of uses. We are confident that this research topic is just at the beginning of its history, and that it will continue to grow, also exploring new fields of application. While the investigation of cellulose–PEI composites as adsorbents of heavy metals and organic contaminants is well established, two other topics are emerging as promising fields of application: the selective sensing of ions and contaminants, and the development of heterogeneous catalysts for an ample spectrum of organic reactions.

What can we learn from this literature review? Could we extract some important suggestions that could drive future developments on this topic?

A first lesson could be derived from the evidence that the choice of the cross-linking strategy seems not to have any effect on the final performance of the resulting cellulose–PEI composites. In contrast to other crucial aspects, for example micro- and nanoporosity or material morphology, the particular selection of a cross-linking procedure seems not to have any significant impact on the results obtained for the specific application. Other criteria could then drive the decision as to which approach should be preferred. The first one is based on the safety and sustainability of the selected cross-linkers or condensing agents, and of the overall synthetic procedure. The second, which is strictly related to the previous one, should consider the chemical and mechanical stability of the final composite, especially in terms of consideration of the main application for which cellulose–PEI composites are proposed, that is, water remediation. In this context, the release of some toxic components, including PEI itself, in the environment, would represent a clear limitation.

These conclusions suggest two additional lessons that can be taken from this brief overview. First, it is recommended that an eco-design approach be followed from the early stage at the laboratory scale for the synthesis of new composites, considering both potential ecotoxicological impacts and the life cycle assessment of the overall process, in order to guarantee sustainability and scalability of final solutions. The second is a natural consequence: new materials should always be tested in real, or at least realistic, scenarios, to effectively exploit the potentialities and limits of the proposed solutions. In too many cases, the scope of the new composite is demonstrated under controlled conditions, such that safety issues and interfering actions of other molecules or ions are dangerously neglected.

## Figures and Tables

**Table 1:** Different types of cellulose used for the synthesis of cellulose–PEI composites.

Cellulose Source	Reference
Cellulose powder	[33]
	[34]
	[35]
	[36]
CMC	[37]
	[38]
CNC	[39]
	[40]
Paper sheets	[41]
	[42]
Medical degrease cotton	[43]

**Table 3:** Principal dye contaminants effectively sorbed by cellulose–PEI composites. The table shows the values of reported sorption capacity for each composite and its reusability.

Dyestuff	Category	Reported Sorption Capacity [mg·g^−1^]	Reusability	Reference
Xilenol Orange	Anionic	241 ^b^	n.d.	[75]
Congo Red		990 ^a^	n.d.	[36]
Reactive Red		950 ^a^	n.d.	[36]
Eosin Y		215 ^a^	n.d.	[36]
Brilliant Blue R		1000 ^a^	n.d.	[36]
		229 ± 7 ^b^	Yes	[76]
Anionic Reactive Yellow		971 ^b^	n.d.	[33]
Naphtol Blue Black		240 ± 10 ^b^	Yes	[76]
Indigo Carmine		540	Yes	[76]
Orange Sodium Salt II		898 ± 16 ^b^	Yes	[76]
Cibacron brilliant Yellow		310	Yes	[76]
Ponceau S		220 ^a^	Yes	[35]
Methyl Blue		1200 ^a^	Yes	[35]
Rose Bengal		1290 ^a^	Yes	[35]
Alizarin Red		360 ^a^	Yes	[35]
Acid Orange 7		280 ^a^	Yes	[35]
Cationic Basic Yellow	Cationic	970 ^a^	n.d.	[36]
Cationic Bright Yellow		160 ^a^	n.d.	[36]
		571 ^b^	n.d.	[33]
Methylene Blue		144 ^b^	n.d.	[75]
		6 ^a^	Yes	[35]

^a^ Data extrapolated from graphs reported in the reference. ^b^ Maximum adsorption capacity calculated exploiting Langmuir isotherm. n.d. means “not determined”.
